# A digital SIW-slot antenna array with FPGA implementation of beamforming

**DOI:** 10.1038/s41598-022-12804-2

**Published:** 2022-05-27

**Authors:** Haitao Li, Shanshan Li, Bo Hou, Xianli Zhang, Weijia Wen, Chuandeng Hu

**Affiliations:** 1 School of Physical Science & Technology and Collaborative Innovation Center of Suzhou Nano Science and Technology, Soochow University, Suzhou, 215006 China; 2 Shenzhen Fantwave Tech. Co., Ltd, Shenzhen, 518110 China; 3 HKUST Shenzhen-Hong Kong Collaborative Innovation Research Institute, Futian, 518000 Shenzhen China; 4 Key Laboratory of Modern Optical Technologies of Ministry of Education and Key Lab of Advanced Optical Manufacturing Technologies of Jiangsu Province, Suzhou, 215006 China; 5 Department of Physics, The Hong Kong University of Science and Technology, Clear Water Bay, Kowloon, Hong Kong, 999077 China

**Keywords:** Engineering, Electrical and electronic engineering

## Abstract

The future satellite platform and 5G communication systems place high demands on antennas, in which the antenna should offer low-cost, lightweight, electronically steerable features. In this paper, the design of a digital slot antenna element based on substrate integrated waveguide (SIW) is proposed. SIW guides the microwave inside the substrate confined with planar metallic covers and through-hole synthetized side-walls in conventional applications, and can also radiate the microwave towards free space in antenna applications through opening slots in its metallic covers. The slot antenna element is realized by implementing PIN diodes across the gaps on both sides of the pad in the center of the slot antenna, to provide the switching freedom of the slot antenna element between radiating and non-radiating states. Besides, radial decoupling stubs are introduced into the bias line so as to reduce the leakage of the energy in the SIW structure. Applying a series of on/off states to the diodes produces various radiation patterns, thus wide range scanning is possible supposing that enough array elements are equipped. Finally, a digital SIW-slot array composed of 8 by 4 elements with tunable field programmable gate array circuits are fabricated and measured. The measured results validate the reconfigurable characteristics for the radiation pattern of the proposed digital SIW-slot antenna array without heavy engineering of phase shifter in conventional antenna arrays. The antenna is consisted by 4 by 8 elements and its dimension, simulated gain and radiation efficiency are 145 mm $\times$ 127 mm $\times$ 1.524 mm, 15 dBi and 53.5%, respectively. Our designed SIW antenna has the advantage of both size and weight. Furthermore, its digitalized control of beamforming allows a programming-friendly interface for smart antenna development.

## Introduction

Typically, traditional phased arrays can achieve $\pm 50^\circ$ cone scanning with the cost of the decrease of gain, except that, active reflection coefficient also deteriorates because of the element mutual coupling^1,2^. Moreover, phase shifters and T/R modules further increase volume and manufacturing costs^3–5^, which makes traditional phased arrays difficult for applying to the mass market.

To overcome those drawbacks of traditional phased arrays, various types of electronically reconfigurable reflectarrays^6–9^ have been reported. In these cases, reflecting elements forms the planar reflectarray aperture and a horn antenna is usually used for feeding. Reflecting elements in Refs.^6,7^ contain liquid crystal, whose dielectric constant changes in different bias voltage, while diodes are equipped in^8,9^. All these methods could change the elements’ reflection phase with variable bias voltage, thus realizing reconfigurable beam. Nevertheless, the distance between feed antenna and reflector arrays is considerable, and it is difficult to integrate them.

In recent years, dynamic metasurface antennas (DMAs) developed rapidly and applied in satellite communication^10,11^, synthetic aperture radar^12–14^, coding metasurfaces^15,16^, metasurface imager^17^ and so on. The traditional DMA is composed of a planar array with many tunable resonant elements evenly placed at equal intervals and fed by the TE10 mode wave propagating in a rectangular waveguide beneath the elements^10^. Compared with the reflectarrays, their feed structures are integrated with radiated antenna elements, which makes the whole structure more compact. Within this structure, each element influences the amplitude and phase of guided wave at each position, and with discrete-dipole approximation (DDA)^18–20^, it is possible in search of good control patterns.

In these studies, complementary electric LC (cELC) resonators were generally adopted as the radiation elements. For instance, Ref.^21^ presents a tunable cELC resonator integrated into a microstrip line, where PIN diodes are introduced to control the on and off characteristic of each cELC element. Though the metamaterial elements indeed reduce the size of array, for example, element spacing is only one fifth of a wavelength in Ref.^11^. However, the entire radiation efficiency is relatively low such as Ref.^13^. Differing from aforementioned, we use digital SIW-slot antenna^22,23^ with relatively high radiation efficiency as the radiation element and investigate the performance of switched beam in the case of array.

SIW has been regarded a promising technique in reducing the footprint of wireless devices, creating the low-profile antenna, and integrating millimeter-wave and terahertz systems^24^, because the substrate can be functioned simultaneously as the waveguide with planar metallic covers and through-hole synthetized side-walls. As the carrier of the radiation elements, SIW structure is generally used in the area of planar antennas including planar array antennas^22,25^. The structured side-wall in SIW is also used as the isolation between the radiating elements in the array antenna^23^. If the radiation elements of the antenna are the slot type, the antenna can be considered as the SIW-slot antenna. Each radiation element can be turned on or off through integrating PIN diodes^26,27^. And the main lobe direction of the antenna is varied with different permutation of on/off status of each radiation element, and this process is also the realization of beam scanning. In short, the antenna in our work can be considered as a kind of digital SIW-slot antenna. This paper is organized as follows. “The process of antenna element design based on SIW” section describes the design process of the tunable SIW-slot antenna array in detail. “Fabrication and measurement results” section presents the fabrication and measurement results of prototype with FPGA circuits and devices. Finally, conclusions are drawn in “Conclusion” section.

## The process of antenna element design based on SIW

**Figure 1:**
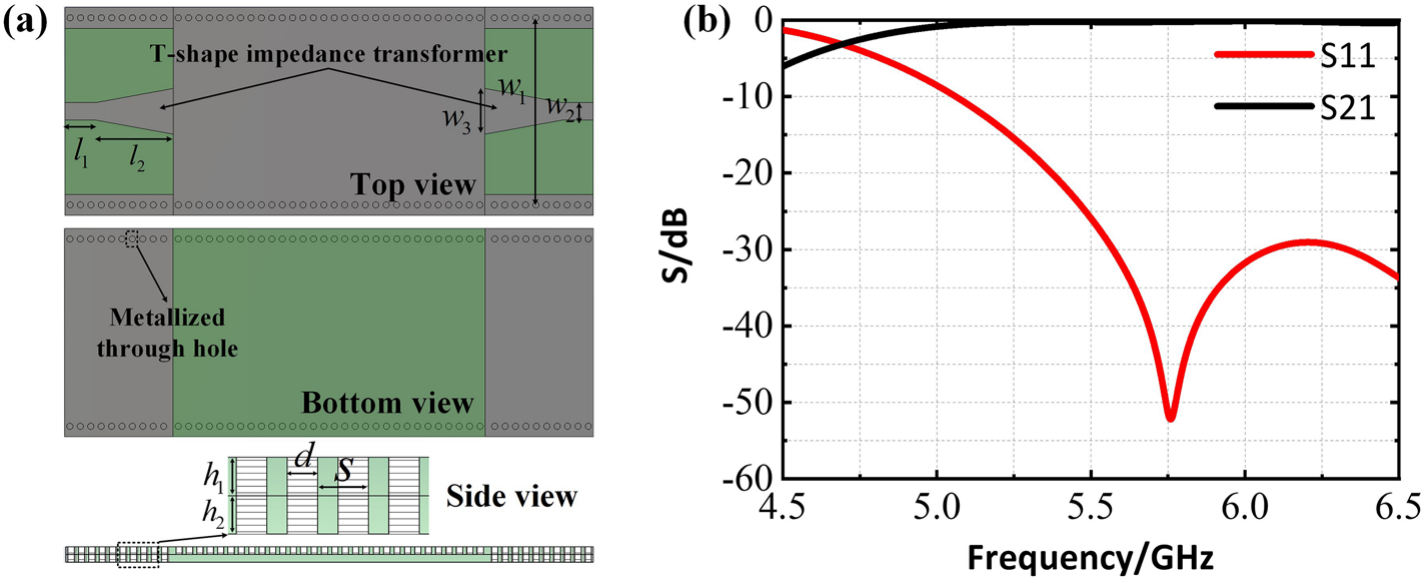
(**a**) Three views of the SIW structure. Some key parameters (length in mm) are: $h_1=0.762$, $h_2=0.762$, $w_1=17.6$, $w_2=1.7$, $w_3=4.4$, $l_1=2$, $l_2=7$, *s* = 1, *d* = 0.3. (**b**) Scattering parameters of the SIW structure.

First of all, a SIW structure (shown in Fig. 1a) is designed with reference to Ref.^28^. Then, a SIW-based four elements slot array is constructed in which the PIN diodes are introduced to guarantee each element can be switched between radiating and non-radiating states. Finally, a digital SIW-slot array composed of 8 by 4 elements is established, where radial decoupling stubs are utilized to prevent energy from leaking to the FPGA circuits and devices.

**Figure 2:**
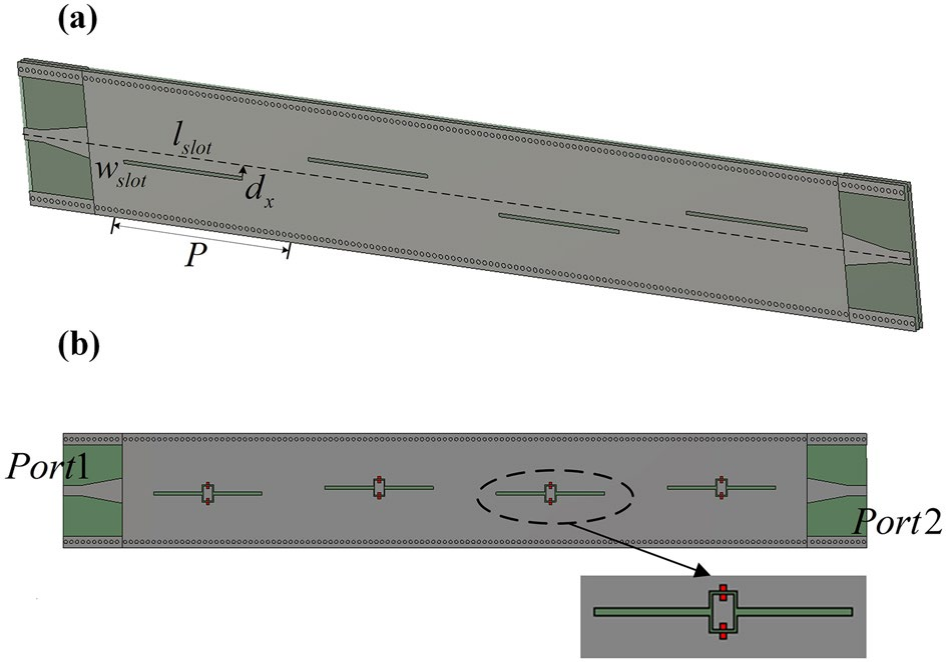
(**a**) The general view of a SIW-based slot antenna without on/off characteristics. Some optimized key parameters (length in mm) are: $w_{slot}=0.5$, $l_{slot}=18.4$, $d_x=0.5$, *P* = 30. (**b**) SIW-based slot array with ideal on/off characteristics.

### Design of a SIW structure

In this subsection, a SIW structure with microstrip-to-SIW transition is optimized (see in Fig. 1a), where two Roger 4350B dielectric plates ( $\epsilon _r$ = 3.66, tan$\delta$ = 0.0037 ) are adopted, serving as support for SIW and tunable circuits and devices respectively, and they are laminated together with a piece of prepreg. Our SIW structure will be fed via SMA connector with the 50$\Omega$ coaxial. Because the physical size of the SMA connector is smaller than the width, $w_1$ of the SIW, an impedance mismatch exists between the SMA and the SIW. The taper-shaped line has been broadly utilized as impedance transformer in microstrip design, because it embodies a smoothing transition between the impedances^29^. Therefore, two sections of taper-shaped microstrips are used to overcome the mismatch at the input and output sides of the SIW. In addition, rectangular patches are printed on the back of the multilayer substrate, which is connected to the SIW structure via metallic through holes. Figure 1b is the simulated results of the SIW structure, which shows satisfactory matching performance and insertion loss in entire WiFi-5G band.

### Four-element slot array

Sequentially, a SIW-based slot array with four identical elements is constructed for the convenience of optimization and quick validation. The performance of SIW slot array^30^ could be dependently adjusted through several dimensions shown in Fig. 2a and the optimization procedure for the radiation efficiency is applied, mainly about the positions of the slot, to achieve the good performance in desired frequency band.

**Figure 3:**
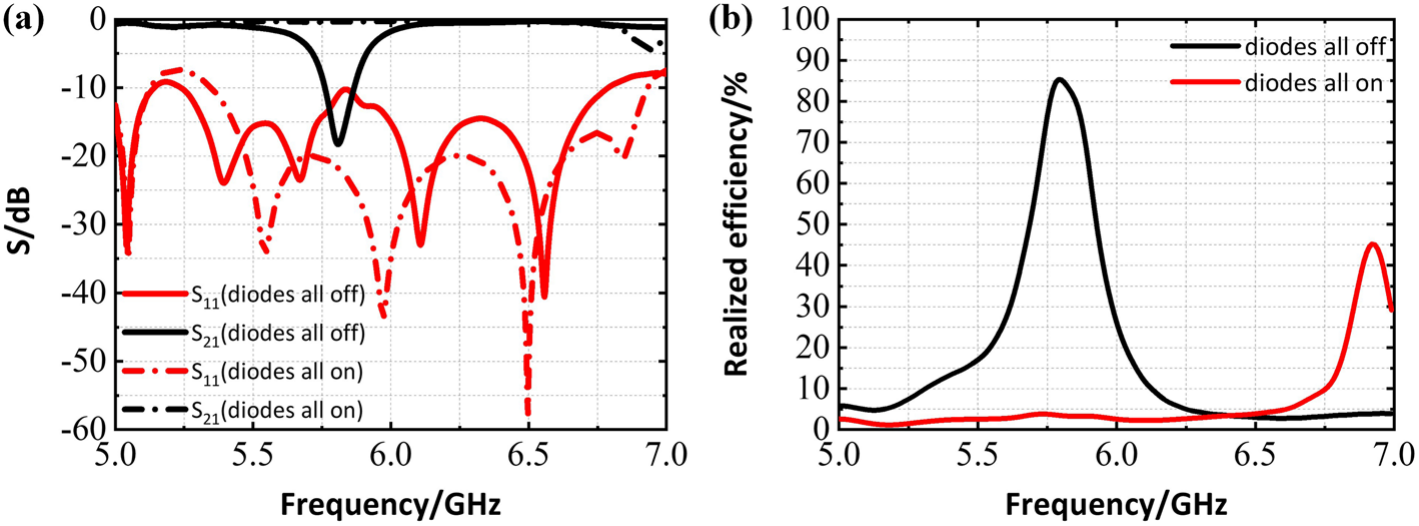
Simulated performance of the designed SIW-based slot antenna (four slot elements) with diodes (All on means diodes are in the on state, all off indicates the opposite) (**a**) Scattering parameters. (**b**) Realized radiation efficiency.

**Figure 4:**
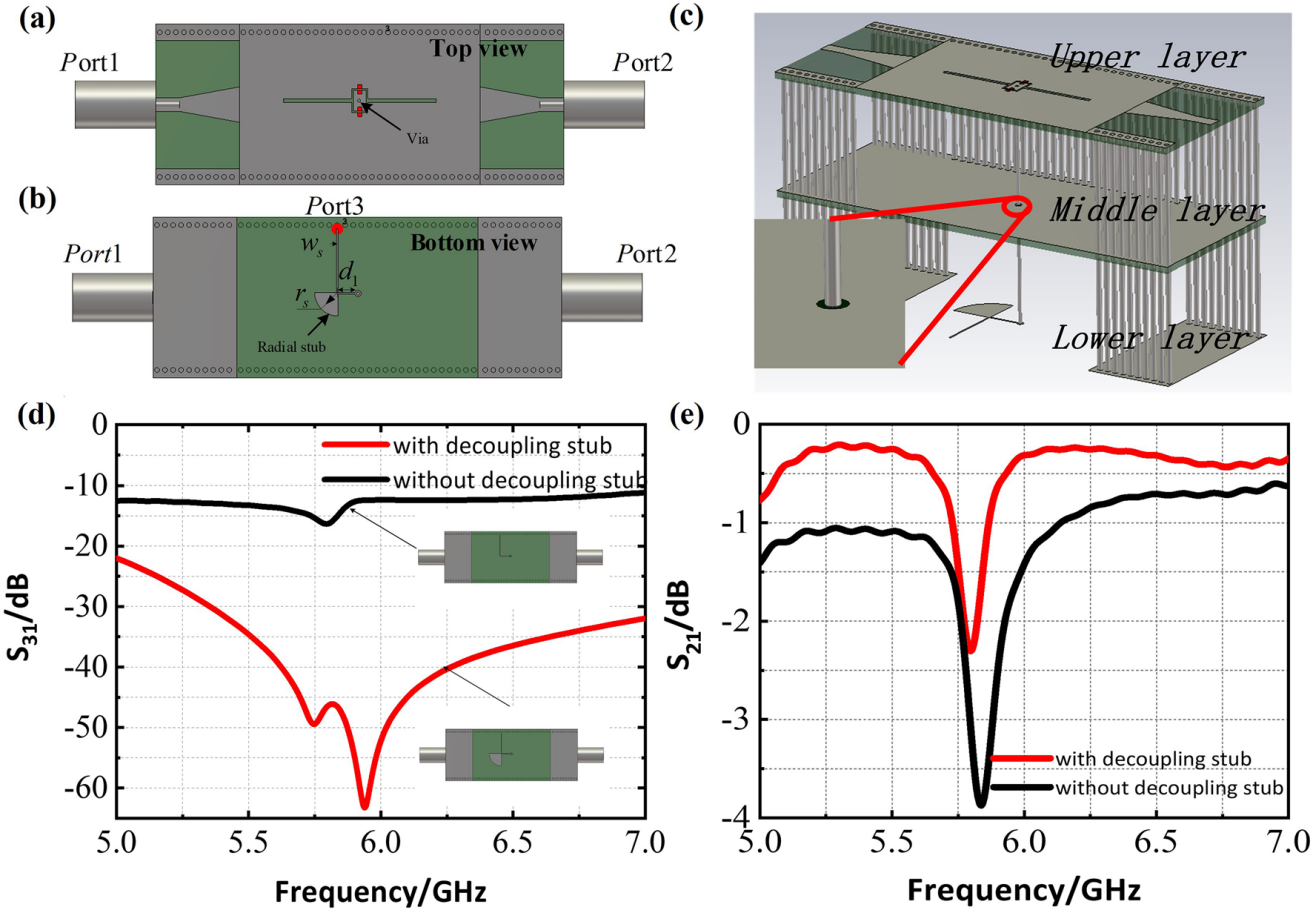
2D sketch of slot element with the radial stub. (**a**) Front view. (**b**) Bottom view. Some optimized key parameters (length in mm) are: $w_s=0.2$, $d_1=4$, $r_s=4.7$. (**c**) Exploded view of the SIW structure with the decoupling stub, the insert indicates that the metallic via is insulated from the ground. (**d**) Scattering parameters with Port-1 at end of the coaxial line and Port-3 connected to the end of DC bias line with and without radial decoupling stub. (**e**) Scattering parameters between Port-1 and Port-2 of the SIW structure with (red solid line) and without (black solid line) radial decoupling stub.

Then, lumped resistors are used to simply imitate the on-off performance of the PIN diodes which are connected across the pads. These resistors are modeled as $R=10^9 \Omega$ and $0 \Omega$ to coarsely simulate off and on state of PIN diodes respectively. The simulated performance of the four elements slot array shown in Fig. 2b are plotted in Fig. 3, where the diodes are in their reverse and forward bias states. It is noted that the slots in Fig. 4 are different from those in Fig. 3 because the pad of diodes (the red parts shown in Fig. 2b) is necessary in experiments, and these pads are nearly no effect on performance of the slot elements. It is seen that when all diodes are in off state, S21 reaches the lowest value at 5.8GHz, meaning that most of the energy radiates through the slots. On the other hand, S21 magnitude is almost 0dB which indicates that the slot array is in the cut-off state when all diodes are in on state. That is to say, when a bias voltage is applied to the PIN diodes in the fabricated device (see the following), the resonance of the shorted slot shifts to higher frequency, thus in-band radiation blocking property is realized, which indicates the realization of digital slot element.

### Tunable SIW-slot antenna array

On the basis of the above design, DC bias lines are introduced to apply voltage to the diodes, and they consist of radial decoupling stubs and metallic vias, as shown in Fig. 4a–c. Due to biasing diodes, the SIW antenna are designed as a multilayer structure in order to print the decoupling stubs on the bottom layer. Metallic vias are introduced for the purpose of the electrical connection between the pads placed on the top layer and the decoupling stubs placed on the bottom layer and the vias are insulated from the ground as shown in the Fig. 4c. The decoupling stubs are used to alleviate the leakage of radio-frequency (RF) electromagnetic energy into the DC biasing circuitry, and otherwise the antenna performance would be much degraded. Therefore, a high isolation need be guaranteed between the RF side (SIW-slot antennas) and the DC side (PIN biasing circuit). The biasing network has been intensively studied and used in microwave amplifiers. In microstrip circuits, a quarter-wave stub or quarter-wave impedance transformer is widely designed to play the isolating role, because it creates a RF short that is a DC open at the same time^29^. In practice, the radial stub is often employed due to its low-impedance feature from the fan-shape and the resulting broadband performance^21,31^. In our design, one of key parameters, $r_s$, is optimized to be 4.7mm that is approximately one quarter of wavelength for 6.0GHz microwave in PCB.

In order to examine the isolation or the decoupling effect, we need calculate any RF energy leaked along the DC bias line. Considering the symmetric layout of the SIW-slot antenna, we choose to locate the third port in the middle of the structure to prevent any coupling of the DC circuit to the RF elements including the input/output ports. As shown in Fig. 4b, we set Port-3 at the upper site of bias line so as to test the performance of the decoupling. In Fig. 4d, the magnitude of S31 represents the severity of energy leakage from SIW structure to the bias circuitry. Besides, Fig. 4e shows the S21 comparison of the SIW structure with (red solid line) and without (black solid line) stub. It is evident that strong energy couples to the bias line without the stub, while the radial decoupling stub effectively reduces the leakage compared with the former. Therefore, our designed decoupling stub plays a role of ‘bias TEE’ between the RF source and the bias circuitry, and allow to integrate the tunable controlling circuits with each slot element and to realize element-specific electrical control for them.

**Figure 5:**
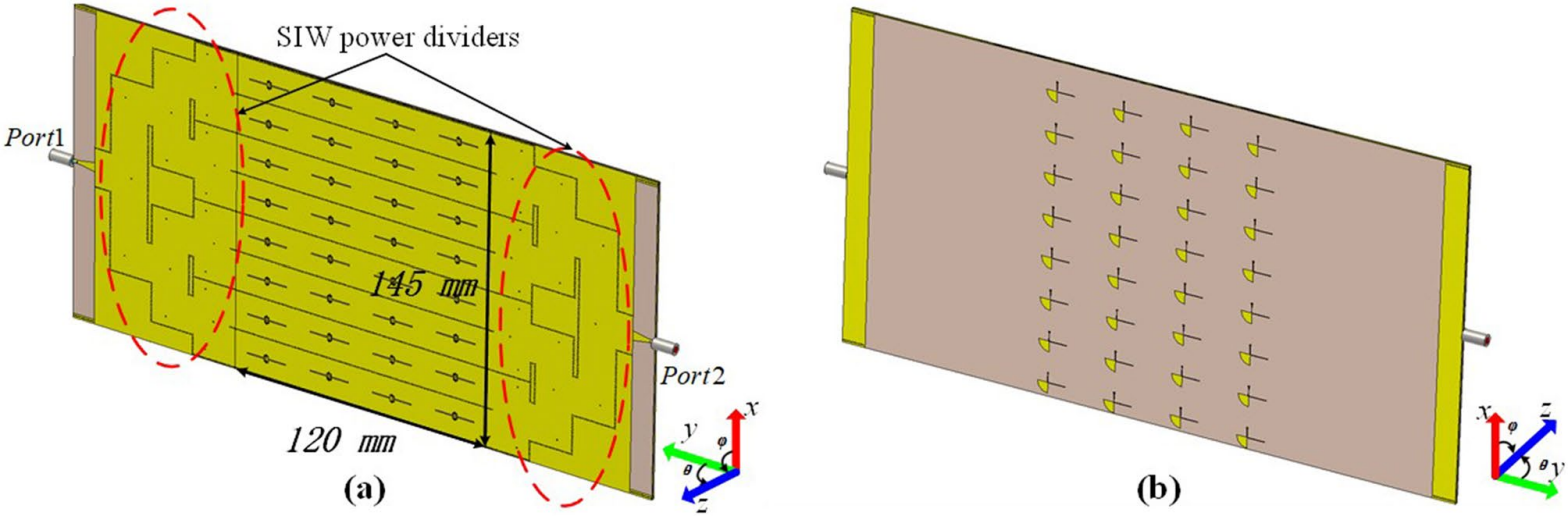
3D schematic of SIW-slot antenna array. (**a**) Front view. (**b**) Bottom view.

**Figure 6:**
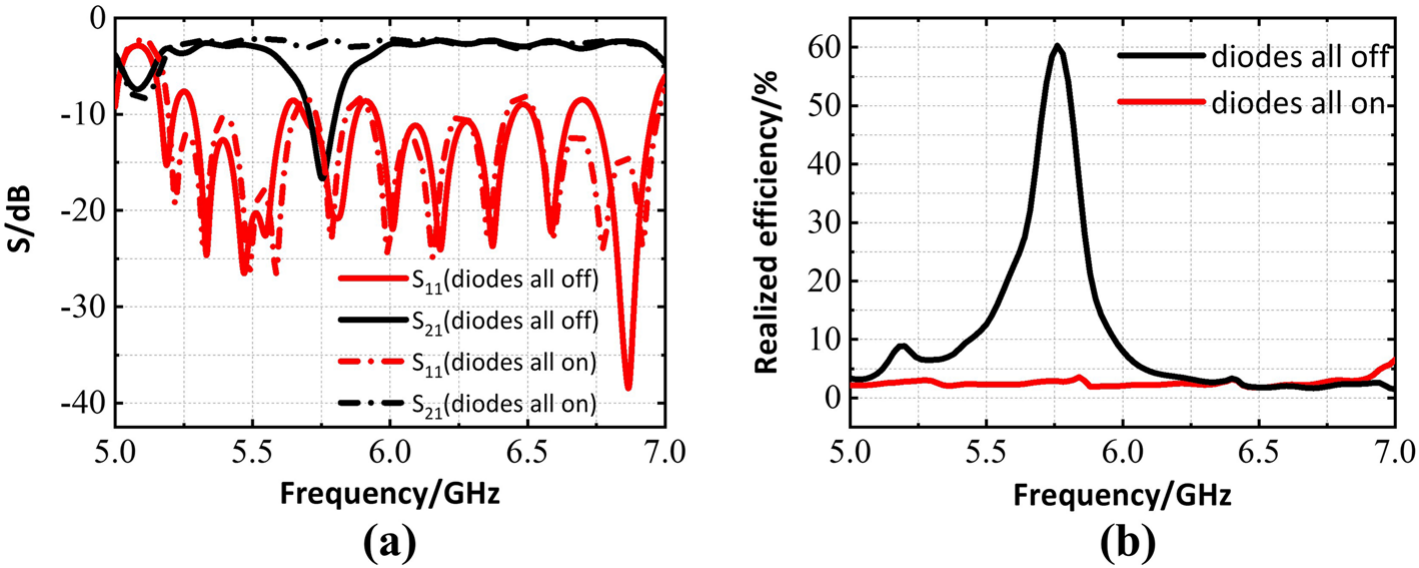
Simulated performance of the designed SIW-slot antenna array (8 by 4 slot elements) (**a**) Scattering parameters. (**b**) Realized radiation efficiency.

In consideration of high isolation between excited port and radial decoupling stubs, and the fact that tunable circuits and devices are placed on the back of antenna which has ignorable effect for entire array, we expand the above 4 elements model to a panel antenna structure which includes 8 by 4 elements and is shown in Fig. 5. The SIW power dividers are applied and optimized for the integration with the slot array. As denoted in Fig. 5a, each of two power dividers adapts a classical T-junction design, where the densely distributed through-holes define the side-walls of the T-junction^32–34^. It is seen that the cascade of three T-junctions divides the power into $2^{3}$ equal parts at the input side and combines them into one channel at the output side, as depicted in Fig. 5a. The back of antenna exists such decoupling stubs, and each of them can individually control the on/off state of the corresponding slot antenna element by changing the bias voltage that is applied to the diodes. In simulation, the change in the state of the diode are imitated by setting the lumped resistance for each diode, as done in Figs. 3 and 6. In practical sample, each stub is supplied by a lead which is connecting with an individual signal channel in the FPGA controlling unit, as shown in Fig. 8b.

The performance of proposed full structure array antenna is shown in Fig. 6, it is clear that our antenna could operate around 5.8 GHz when diodes are in off state, while applying forward bias voltage to all diodes can stop energy radiating from the slots within operating band, it is similar with aforementioned four elements slot array. At the same time, the efficiency decreases on account of the power division network and dielectric loss. However, it is passable compared with the cases in Refs.^12–14^.

**Figure 7:**
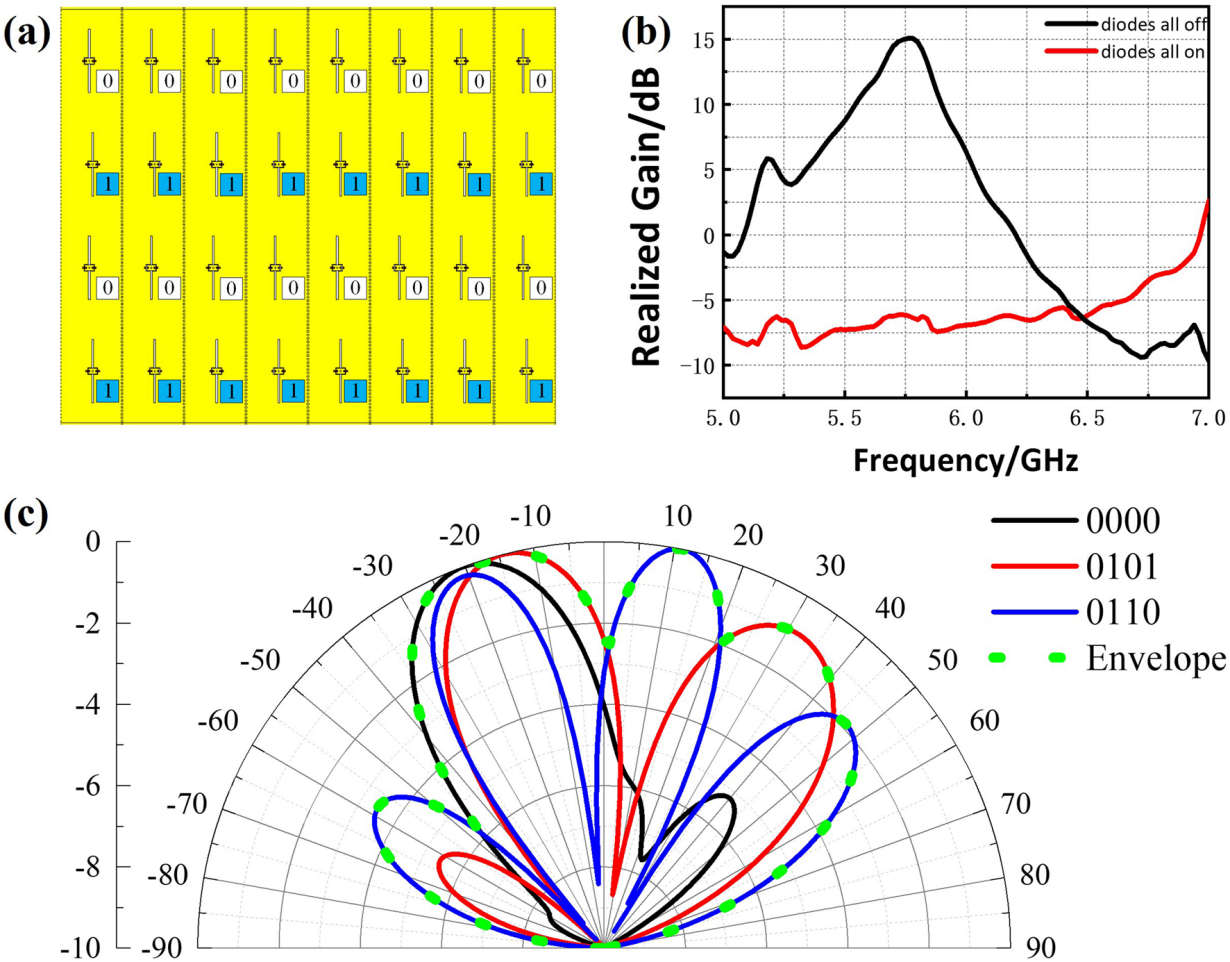
(**a**) Binary coding for bias voltage distributions (‘0101’ state as example). (**b**) Simulated realized gain of both ‘0000’ and ‘1111’ states. (**c**) Simulated far field normalized radiation patterns of the tunable designed SIW-slot antenna array in several on/off states of diodes ($\varphi =90^\circ$).

Furthermore, the array can be tuned to generate various complex patterns once diverse bias voltages are applied to the diodes. Hence, we define 1/0 as high/low level, which correspond to the on/off state of diodes. For convenience’s sake, 0/1 sequences change along the direction of propagation of the electromagnetic wave in the SIW structure and keep the same along the direction orthogonal to the above one, which is expressed more clearly in Fig. 7a.

Here, three typical binary sequences are selected to simulate on CST Microwave Studio, and the realized gain for diodes on/off and normalized radiation patterns at the center frequency of 5.8 GHz are plotted in Fig. 7b,c, respectively, from which we can intuitively see the switched beams under different sequences, which acts like a phased array, though the lacking of the T/R modules.

Theoretically speaking, the sequence of ‘0101’ or ‘0110’ is corresponding larger spacing between two radiated elements compared with ‘0000’ one, meaning that more lobes would emerge, which is consistent with the simulation results. A beam envelope plotted in green dot could be extracted from these patterns which represents the dynamic range for beam scanning that can be controlled under current sequences set. It is deduced that the binary sequences vary in two dimensions would introduce more degree of freedom, thus readily realizing arbitrary beam synthesis in a wide region of solid angle combined with DDA method as long as there are enough tunable elements according to Ref.^18^.

## Fabrication and measurement results

**Figure 8:**
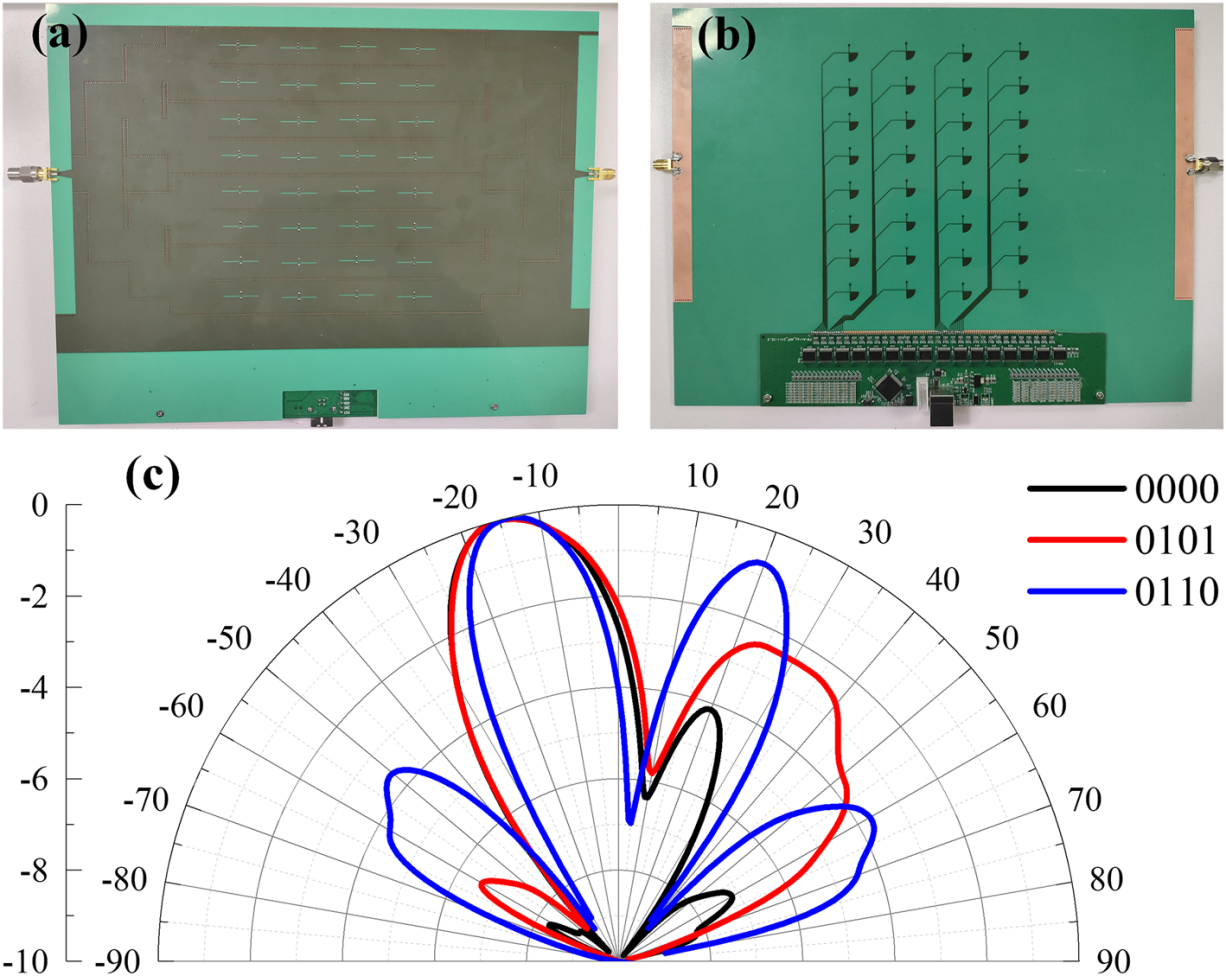
Overall view of the assembled tunable SIW-slot antenna array and the corresponding measured results. (**a**) Front view of the antenna. (**b**) Bottom view of the antenna. (**c**) Measured far field normalized radiation patterns of the tunable designed SIW-slot antenna array in several on-off states of diodes ($\varphi =90^\circ$).

As shown in Fig. 8a,b, the digital SIW-slot antenna array prototype with FPGA circuits is fabricated and assembled. The FPGA board is designed and customized for the antenna array, and its position in the prototype is located away from the SIW region, seeing the bottom of Fig. 8a,b. It is attached on the antenna by its 32 pins welding to each PIN bias feeder connecting to the decoupling stub, as shown in Fig. 8b. A small section (PCB part) of the antenna board has been cut to accommodate the extruding circuit elements on the FPGA board, seeing Fig. 8a. Finally, the FPGA board are secured together with the planar antenna array by metal screws.

The far-field pattern measurements are conducted in an anechoic chamber. The measured antenna is fixed at a turntable to receive the far-field power, and a wideband ridged horn antenna operating over 1-16GHz is used for transmitting antenna. A personal computer is connected to the serial port on the FPGA circuit board, thus each element of array is individually controlled. Figure 8c shows the measured normalized radiation patterns of the fabricated antenna array in several states at the center frequency of 5.6 GHz. Though some discrepancies of center frequency occur due to diodes with complex equivalent circuit model, besides, the fabrication and assembly process also introduce some unknowns, experimental results still reach a good agreement with simulated results.

The appealing features of our antenna lie in its low-profiled simple structure and FPGA-friendly programming interface. In our perspective, programming antenna will hold a bright future, given the rapid development of both 5G/6G high-speed wireless communication and information technologies, e.g. artificial intelligence, in now days. At the bottom of the paper, the comparison of some indexes among several references with this work is listed in Table 1. Although the antenna we demonstrated in this work is not very outstanding among these works, the performance could be improved by optimization, e.g., larger array.

**Table 1:** Comparison of some indexes among several references with this work.

References	Antenna	Dimensions ($\mathrm{mm}^{3}$)	Frequency (GHz)	Bandwidth (GHz)	Gain (dBi)	Efficiency ($\%$)	Beam characteristics
Ref.^23^	SIW-loaded patch array	20 × 13.5 × 0.125	190–200	10	Max 12.2	Max 86	Static beam
Ref.^22^	MTM- and SIW-inspired Bowtie Antenna	30 × 16 × 0.8	30–37	7	5.5	66.5	Static beam
Ref.^27^	A Simple Compact Reconfigurable Slot Antenna	35 × 20 × 1.864	0.42–1.48	1.06	Above –0.4	–	Static beam
Ref.^26^	SIW antenna array	75 × 61 × 1.575	2.45	0.08	3$\sim$6	–	Switched beam
Ref.^33^	SIW Vivaldi antenna array	85 × 70 × 1.6	8.88–10.02	1.14	13.3	–	Static beam
Ref.^6^	Reflect array	574 × 574 × 0.254	60.25	0.45	42	9.5	Highly directive beam, electronical steering
Ref.^21^*	Waveguide-fed aperture array	–	$\sim$21.5	–	–	38	Directive or diverse beams electronical tuning
This work	SIW slot antenna array	145 × 120 × 1.524	5.75	0.1053(– 10dB)	15	53.5	FPGA controlled tunable beams

*Denotes that only the simulation results are included in the reference.

–Denotes that this index is missing in the reference.

## Conclusion

Traditional phased arrays suffer from some disadvantage of heavy and expensive, in other words, some hardware such as phase shifters and T/R modules, thus usually applied to military rather than commercial use. This paper presents the design, fabrication and measurement of a digital SIW-slot antenna array with PIN diodes and tunable FPGA circuits, which can achieve considerable performance compared with phased arrays and mechanically scanned systems. Measured results verified that the arbitrary radiation patterns synthesis of the tunable SIW-slot array is potential. The simulated max realized gain is 15dB at the operate frequency, and the radiation efficiency reach the highest point 53.5%. The radiation performance can be improved through implementing larger array. Although only patterns in several on/off sequences of diodes are measured for proposed antenna, accurate and efficient methods for diverse, highly directive and complex radiation patterns can also be efficiently synthesized by applying the DDA method as mentioned above in larger array.

## Data Availability

The datasets generated and/or analysed during the current study are not publicly available due to the patent filing but are available from the corresponding author on reasonable request.
